# Curcumin improves memory deficits by inhibiting HMGB1‐RAGE/TLR4‐NF‐κB signalling pathway in APPswe/PS1dE9 transgenic mice hippocampus

**DOI:** 10.1111/jcmm.16855

**Published:** 2021-08-18

**Authors:** Yuan Han, Rui Chen, Qicheng Lin, Yu Liu, Wenwei Ge, Hong Cao, Jun Li

**Affiliations:** ^1^ Department of Anesthesiology and Perioperative Medicine The Second Affiliated Hospital and Yuying Children’s Hospital of Wenzhou Medical University Wenzhou China; ^2^ Zhejiang Province Key Laboratory of Anesthesiology Wenzhou Medical University Wenzhou China

**Keywords:** advanced glycosylation end product‐specific receptor, Curcumin, HMGB1 protein, nuclear factor kappa B, Toll‐like receptor 4

## Abstract

Amyloid‐β (Aβ) deposition in the brain has been implicated in the development of Alzheimer's disease (AD), and neuroinflammation generates AD progression. Therapeutic effects of anti‐inflammatory approaches in AD are still under investigation. Curcumin, a potent anti‐inflammatory and antioxidant, has demonstrated therapeutic potential in AD models. However, curcumin's anti‐inflammatory molecular mechanisms and its associated cognitive impairment mechanisms in AD remain unclear. The high‐mobility group box‐1 protein (HMGB1) participates in the regulation of neuroinflammation. Herein, we attempted to evaluate the anti‐inflammatory effects of chronic oral administration of curcumin and HMGB1 expression in APP/PS1 transgenic mice AD model. We found that transgenic mice treated with a curcumin diet had shorter escape latencies and showed a significant increase in percent alternation, when compared with transgenic mice, in the Morris water maze and Y‐maze tests. Additionally, curcumin treatment could effectively decrease HMGB1 protein expression, advanced glycosylation end product‐specific receptor (RAGE), Toll‐like receptors‐4 (TLR4) and nuclear factor kappa B (NF‐κB) in transgenic mice hippocampus. However, amyloid plaques detected with thioflavin‐S staining in transgenic mice hippocampus were not affected by curcumin treatment. In contrast, curcumin significantly decreased GFAP‐positive cells, as assessed by immunofluorescence staining. Taken together, these data indicate that oral administration of curcumin may be a promising agent to attenuate memory deterioration in AD mice, probably inhibiting the HMGB1‐RAGE/TLR4‐NF‐κB inflammatory signalling pathway.

## 1 INTRODUCTION

Alzheimer's disease (AD) is the most common form of dementia worldwide and is characterized by its impact on cognition, problem‐solving and other skills that affect a person's daily life.^1^, ^2^ Neurofibrillary tangles and abnormal accumulation of amyloid‐β (Aβ) could trigger astrocytes and microglial cell activation, leading to neuroinflammation, which may generate AD progression.^3^, ^4^, ^5^ The hippocampus, which plays an important role in memory and cognition, is vulnerable to damage or destruction by neuroinflammation. Therefore, modulation of neuroinflammation progression might be a promising treatment to ameliorate AD progression. APP/PS1 transgenic mice are a widely used mouse model for AD disease.^6^

Extracellular high‐mobility group box‐1 protein (HMGB1) levels are elevated in the brains of patients with AD.^7^ HMGB1, a non‐histone nuclear protein, serves as a damage‐associated molecular pattern that activates sterile inflammation signal amplification through a variety of receptors, including advanced glycosylation end product‐specific receptor (RAGE) and Toll‐like receptors (TLRs), which are involved in the activation of the NF‐κB signalling pathway.^8^, ^9^ HMGB1 is also associated with several other molecules, such as chemokine CXC ligand 12 (CXCL12),^10^ CD24,^11^ interleukin‐1β (IL‐1β)^12^ and nucleosomes.^9^ RAGE is a cell surface multi‐ligand receptor that belongs to the immunoglobulin superfamily, and its expression correlates with the inflammation severity in AD.^13^ During the activation of the innate immune system, pathogen‐associated molecular patterns are recognized by TLRs and type I transmembrane proteins with N‐terminal domain‐containing leucine‐rich repeats. TLR4 expression is increased in both AD transgenic mice and patients with AD, and subsequently elicits inflammatory responses.^14^ NF‐κB, a transcription factor, plays a crucial role in regulating inflammation, stress and immune responses. The aberrant activation of NF‐κB has been associated with various diseases, such as neurodegenerative and autoimmune diseases, diabetes and cancer.^15^

Traditional anti‐Aβ drugs have failed in clinical trials,^16^ and their mainstream treatment targets are cholinesterase inhibitors and N‐methyl‐D‐aspartic acid receptor antagonists.^17^ These drugs usually have strong side effects. Therefore, new treatment agents are urgently required. Curcumin, extracted from turmeric rhizomes, is a Sino Food and Drug Administration‐approved food additive in the curry spice turmeric. It possesses potent anti‐inflammatory and antioxidant properties and is widely used to treat chronic inflammatory diseases, such as heart disease, cancer, metabolic syndrome and various other degenerative diseases.^18^ It has therapeutic potential in AD models, both in vitro and in vivo.^19^ The molecular mechanisms involved in curcumin activity in patients with AD remain poorly understood. The present study aimed to evaluate the potential of oral curcumin in preventing memory decline and to explore the anti‐inflammatory molecular mechanisms of curcumin action in AD transgenic mice.

## 2 MATERIALS AND METHODS

### 2.1 *Drugs* *and reagents*

Curcumin was purchased from Cayman Chemical (Ann Arbor, MI, USA). Phosphate‐buffered saline (PBS, pH 7.4), paraformaldehyde (PFA), Triton X‐100 and BCA assay kits were purchased from Thermo Fisher Scientific (Carlsbad, CA, USA). Thioflavin‐S was purchased from Sigma‐Aldrich (St. Louis, MO, USA). Cell lysis buffer and phenylmethylsulfonyl fluoride (PMSF) were purchased from Beyotime Biotechnology (Shanghai, China). Primary antibodies used for immunofluorescence staining and Western blot, including goat polyclonal anti‐GFAP antibody, rabbit polyclonal anti‐COX2 antibody, rabbit monoclonal anti‐HMGB1 antibody, rabbit polyclonal anti‐RAGE antibody, mouse monoclonal anti‐TLR4 antibody, rabbit monoclonal anti‐NF‐κB p65 antibody and mouse polyclonal anti‐β‐actin antibody were purchased from Abcam (Cambridge, UK), Cell Signaling Technology (CST, Danvers, MA, USA) or Bioworld (St. Louis Park, USA). Secondary antibodies, including DyLight 488 AffiniPure Donkey Anti‐Goat IgG, HRP‐labelled rabbit anti‐goat, HRP‐labelled goat anti‐rabbit and HRP‐labelled goat anti‐mouse, were obtained from EarthOx (Millipore, Burlington, MA, USA) or Fdbio Science (Hangzhou, China).

### 2.2 Animals and protocol

Three‐month‐old male B6C3‐Tg (APPswe, PSEN1dE9) 85Dbo/NJU transgenic mice (also known as APP/PS1 transgenic mice) and their wild‐type littermates (Model Animal Research Center of Nanjing University, Nanjing, China) were housed in a pathogen‐free facility with a temperature‐controlled at 22 ± 2°C and 12‐h light/12‐h dark cycle. They had free access to water and food. All animal studies were performed following the guidelines of the Laboratory Animal Ethics Committee of Wenzhou Medical University and Laboratory Animal Center of Wenzhou Medical University (licence: wydw2013‐0025).

Twenty transgenic mice were randomly divided into control transgenic (TG) and curcumin‐treated transgenic (CUR) mice, and the other ten littermate wild‐type (WT) mice served as controls (*n* = 10 per group). CUR mice were fed normal diets containing 750 ppm of curcumin for 5 months, from 4 months of age (100 mg/kg/d), and others were fed a normal diet without curcumin.

**FIGURE 1:**
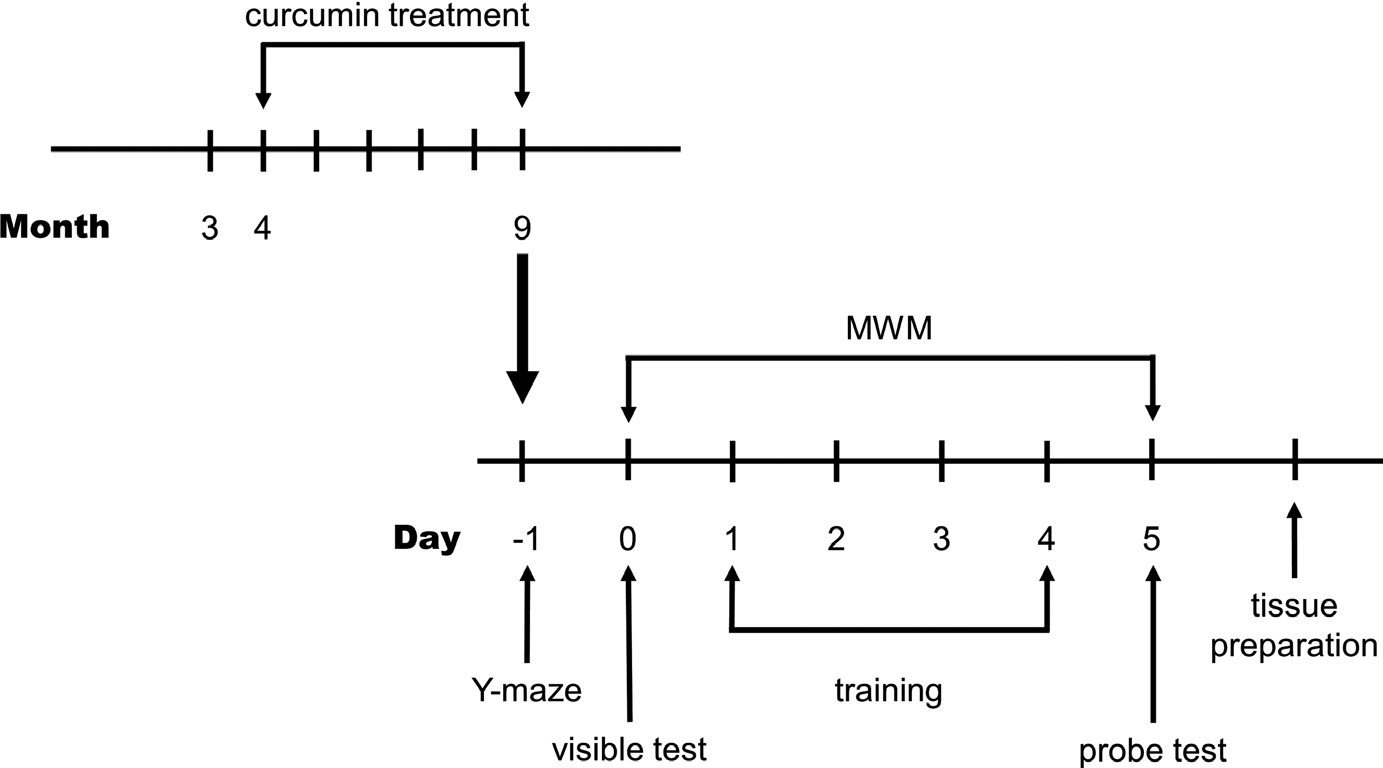
Experimental design to investigate the role of curcumin treatment in APPswe/PS1dE9 transgenic mice. The transgenic mice at 4 months old were chronic oral administration curcumin for five months. All mice were subjected to Y‐maze test on day −1 followed by Morris water maze test. After having completed the behavioural experiments, brain tissues were harvested

The experimental procedures of curcumin treatment, Morris water maze and Y‐maze tests are displayed in Figure 1.

### 2.3 Morris water maze

The Morris water maze (MWM) is one of the most widely used behavioural tasks for assessing learning and reference memory. We conducted the MWM as previously described.^20^ Briefly, the equipment consisted of a large black circular pool (90 cm in diameter, 50 cm in height, filled with opaque white water at 22 ± 1°C). A black escape platform (4.5 cm in diameter) was submerged 1.0 cm below the pool water surface and placed in the centre of the fourth quadrant throughout the study. Opaque white curtains surrounded the pool outside to control the visual cues available to the animal. Each mouse received daily training trials for four consecutive days, with each trial having a 60 s cut‐off time. A mouse was placed in the water at one of four starting positions in each quadrant, the sequence order of which was selected randomly, and the time required for the mouse to find the hidden platform was recorded as the escape latency. A mouse that found the platform was allowed to remain on the platform for 15 s and then returned to its cage for the inter‐trial interval. A mouse that did not find the platform for up to 60 s was placed on the platform for 15 s at the end of the trial.

To evaluate consolidated spatial memory, the platform was removed during the probe trial on the 5th day and each mouse was allowed to search for the platform in the pool for 60 s. Latency to find the platform, the number of times crossing over the platform site of each trial, and the swimming time spent in the target quadrant were recorded. All data were recorded using a computerized video system (SLY‐WMS 2.0, Beijing Sunny Instruments, China).

### 2.4 Y‐maze

Spatial working memory was assessed by spontaneous alternation behaviour in the Y‐maze, as described previously.^21^ The Y‐maze had three identical arms, 35‐cm‐long, 8‐cm‐wide and 15‐cm‐high. Each mouse was placed in the centre of the maze and allowed to explore the arena for 5 min. The number and sequence of arm entries were recorded. The animal's successive entries into the three arms without repetitions were seen as the end of one alternation, and percent alternation was calculated as the ratio of actual alternations divided by total entries minus two. The maze was thoroughly cleaned with ethanol 75% after each session.

### 2.5 Tissue preparation

Animals were anaesthetized with pentobarbital (200 mg/kg, intraperitoneal) and killed by decapitation after the completion of the behavioural experiments. Brain tissues were harvested for further assays. For thioflavin‐S staining and immunofluorescence, animals were transcardially perfused with PBS, followed by 4% PFA. The brain was then removed, post‐fixed in 4% PFA for 6 h, and subsequently dehydrated in 10%, 20% and 30% sucrose solutions at 4°C. The brains were embedded in an optimal cutting temperature compound for sectioning. For Western blot analysis, some cohorts were decapitated, and their hippocampi were dissected out and immediately homogenized in ice‐cold cell lysis buffer containing 1 mM PMSF. The homogenates were sonicated three times, centrifuged at 12,000 rpm for 40 min at 4°C, and the supernatant was collected. The protein was quantified using a BCA assay kit, according to the manufacturer's instructions.

### 2.6 Immunofluorescence staining

To evaluate astrocyte activation, the expression of the glial fibrillary acidic protein (GFAP), a protein in the cytoskeleton of mature astrocytes, was evaluated by immunofluorescence staining, which was performed as previously.^22^ Briefly, frozen sections were rinsed thrice with PBS for 5 min each, treated with 0.3% Triton X‐100 for 10 min, blocked with 10% donkey serum for 2 h and incubated with goat polyclonal anti‐GFAP antibody (1:1000) at room temperature for 1 h or overnight at 4°C. The secondary antibody used was DyLight 488 AffiniPure donkey anti‐goat IgG (1:200). Images were taken using a fluorescence microscope (Nikon, Japan). Quantitative fluorescence intensity was collected from at least three slices per mouse (*n* = 3 per group) randomly, and the average of three readings was used for further analysis. Data were processed and analysed using Image‐Pro Plus 6.0 (Media Cybernetics, Inc., Rockville, MD, USA).

### 2.7 Western blot analysis

Samples were separated by denaturing SDS‐polyacrylamide gel electrophoresis and electrotransferred onto a polyvinylidene difluoride membrane. After blocking with 10% skim milk for 2 h, the membranes were incubated at 4°C overnight with goat polyclonal anti‐GFAP antibody (1:500), rabbit polyclonal anti‐COX2 antibody (1:200), rabbit monoclonal anti‐HMGB1 antibody (1:1000), rabbit polyclonal anti‐RAGE antibody (1:500), mouse monoclonal anti‐TLR4 antibody (1:500), rabbit monoclonal anti‐NF‐κB p65 antibody (1:500) or mouse polyclonal anti‐β‐actin antibody (1:5000). Subsequently, the membrane was washed and incubated with HRP‐labelled rabbit anti‐goat (1:5000), HRP‐labelled goat anti‐rabbit (1:5000) or HRP‐labelled goat anti‐mouse (1:5000) secondary antibodies for 2 h. Immunoreactivity was detected using the SuperSignal West Femto assay kit (Thermo Fisher Scientific, Pierce) according to the manufacturer's instructions. Western blot bands were scanned using Image Quant LAS4000mini (GE Healthcare, Piscataway, USA) and evaluated using densitometry in Quantity‐One 1‐D Analysis Software (Bio‐Rad, Laboratories, Inc., Hercules, CA, USA).

### 2.8 Thioflavin‐S staining

The brain was sectioned in the coronal plane (Microm, Walldorf, Germany) at 20‐μm slice thickness. Thioflavin‐S staining of plaques was performed as previously described^6^ with some modifications. Sections were washed in PBS for 5 min three times and incubated in 1% thioflavin‐S for 10 min, washed in 50% ethanol for 5 min two times, and in PBS for 5 min, and then visualized using fluorescence microscopy (Nikon, Japan). Thioflavin‐S staining data were processed and analysed using Image‐Pro Plus 6.0.

### 2.9 Statistical analysis

Statistical comparison was performed using one‐way analysis of variance (ANOVA) followed by LSD or Tamhane's T2, wherever appropriate. A two‐way repeated‐measures ANOVA was conducted to analyse cognitive performance in the MWM training phases. Data are presented as mean ± standard deviation (SD). All statistical analyses were performed using the SPSS software 21.0 (IBM Corp., Armonk, NY, USA). Statistical significance was set at *p* < 0.05.

## 3 RESULTS

### 3.1 Body weight and motor function

**FIGURE 2:**
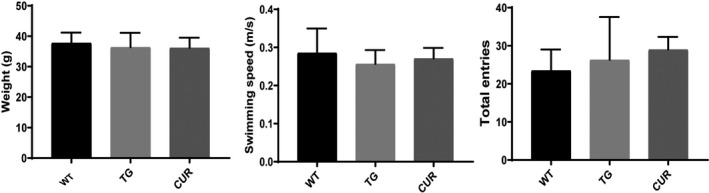
The body weight and motor function of all experimental mice. (A) Body weight, (B) swimming speed performance and (C) total entries of wild‐type (WT), control transgenic (TG) and curcumin‐treated transgenic (CUR) mice. The data are expressed as mean ± SD (*n* = 10 per group)

Mice body weight was not significantly different among groups during the experiments (Figure 2A). The baseline body weight was 36.4 ± 4.7 g, 36.2 ± 3.3 g and 37.8 ± 3.4 g in the TG, CUR and WT groups, respectively. Motor function was assessed by the swimming speed in the MWM test and the total entries in the Y‐maze. As shown in Figure 2B,C, the swimming speed performance (one‐way ANOVA, *p* = 0.373) and the total entries (one‐way ANOVA, *p* = 0.277) were not significantly different between the experimental groups, indicating that the 20 transgenic mice were relatively suitable experimental candidates.

### 3.2 Curcumin improves spatial learning and memory of transgenic mice

**FIGURE 3:**
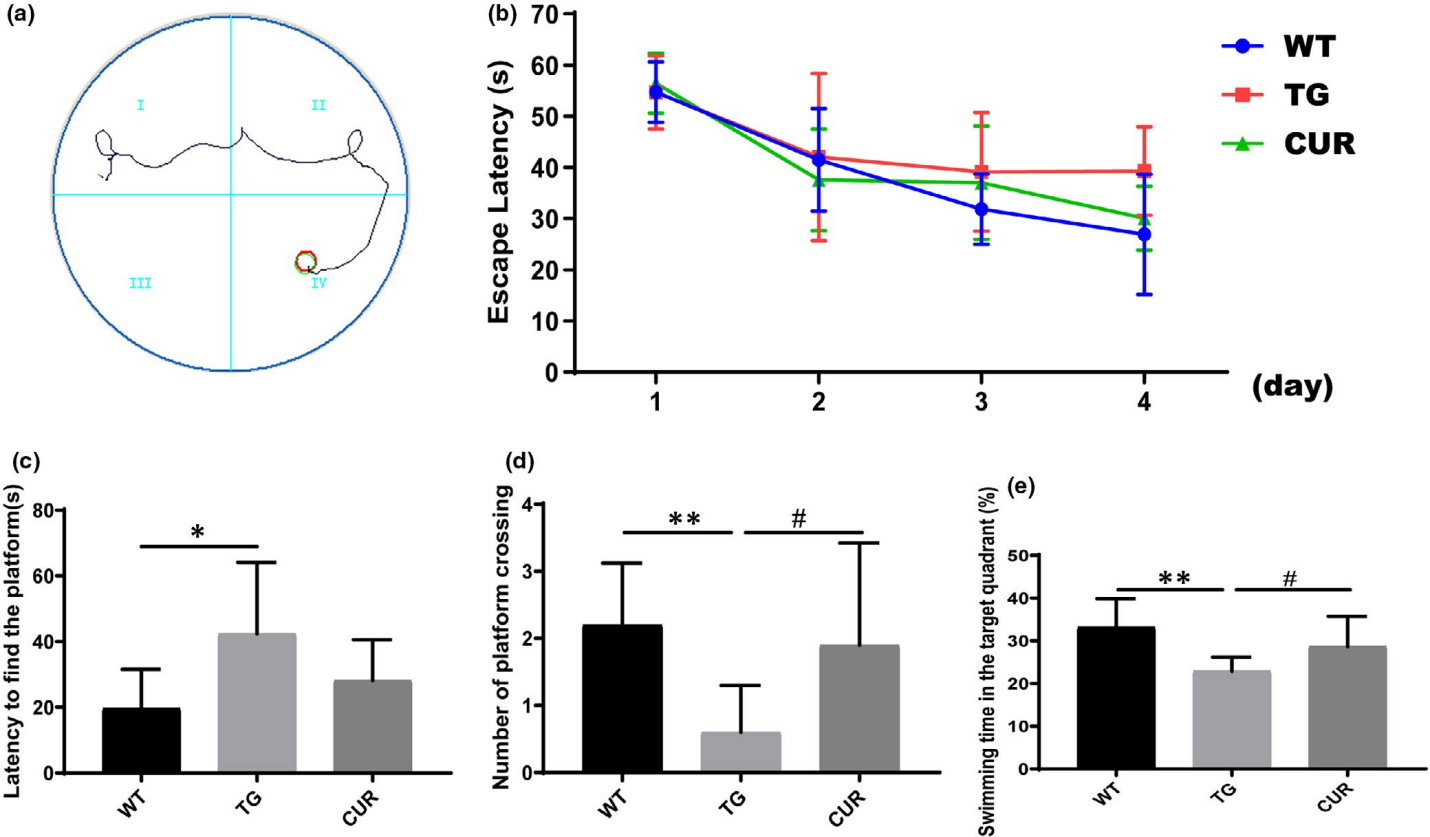
Effect of curcumin on spatial learning and memory of APPswe/PS1dE9 transgenic mice. (A) Mice swimming trajectory in Morris water maze test (MWM) test. (B) Mice escape latency on days 1, 2, 3 and 4 during training phases in the MWM test. (C) The latency to find the submerged platform of all mice on day 5 during probe test in the MWM test. (D) The number cross‐platform of all mice on day 5 during probe test in MWM test. (E) Swimming time in the target quadrant of all mice on day 5 during probe test in MWM test. WT, wild‐type mice; TG, control transgenic mice; CUR, curcumin‐treated transgenic mice. The data are expressed as mean ± SD (*n* = 10 per group), two‐way ANOVA (repeated measure) was performed for training phase analysis and one‐way ANOVA for probe test in MWM test. *p < 0.05 TG vs. WT; **p < 0.01 TG vs WT; ^#^ *p* < 0.05 CUR vs. TG

The three experimental groups underwent the MWM test to determine whether curcumin could improve spatial learning and memory. All animals were allowed to find the submerged platform in an open swimming arena after four days of training, and the platform was removed during the probe trial on the 5th day. The trajectory of the mouse was recorded using a computerized video system, as shown in Figure 3A. All groups of mice showed improvement in their spatial learning and memory performance, as indicated by the decreased escape latencies across successive days, but the CUR group and other ten WT mice performed much better than the TG group (Day 4: WT: 26.94 ± 11.75 s, TG: 39.28 ± 8.67 s, CUR: 30.07 ± 6.26 s, two‐way ANOVA, *p* < 0.001) (Figure 3B). The mice in the TG group displayed cognitive deficits compared with WT, as indicated by the prolonged latency to find the platform (Figure 3C, one‐way ANOVA, *p* = 0.036, from the probe test on day 5), fewer platform crossings (Figure 3D, one‐way ANOVA, *p* = 0.003, from the probe test on day 5) and less time spent in the target quadrant (Figure 3E, one‐way ANOVA, *p* = 0.001 from the probe test on day 5). Moreover, TG mice were attenuated by the oral treatment of curcumin, as indicated by the decreased trend of escape latency (one‐way ANOVA, *p* = 0.256), more number of platform crossings (one‐way ANOVA, *p* = 0.014) and a higher percentage of time spent in the target quadrant (one‐way ANOVA, *p* = 0.037) than the CUR mice.

### 3.3 Curcumin reverses short‐term working memory defects of transgenic mice

**FIGURE 4:**
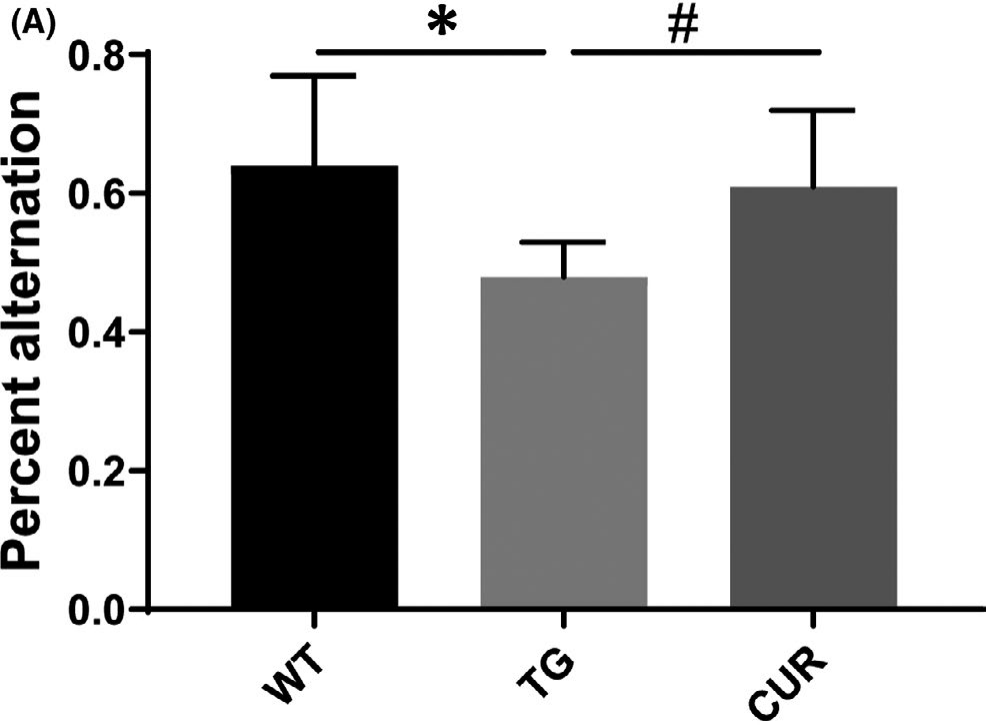
Effect of curcumin on the short‐term working memory of APPswe/PS1dE9 transgenic mice. (A) The percent spontaneous alternation of all mice. WT, wild‐type mice; TG, control transgenic mice; CUR, curcumin‐treated transgenic mice. The data are expressed as mean ± SD (*n* = 10 per group), one‐way ANOVA. *p < 0.05 TG vs. WT; ^#^ *p* < 0.05 CUR vs. TG

Y‐maze tests are sensitive for examining the willingness of rodents to investigate new environments and short‐term working memory. A mouse with good functional working memory will show a lower tendency of repeated entries into the same arm. The percentage of spontaneous alternations in the TG mice was significantly lower than that in the WT group (0.48 ± 0.05 vs. 0.64 ± 0.13, one‐way ANOVA, *p* = 0.013), whereas curcumin treatment increased the percent alternation of TG mice (0.61 ± 0.11, one‐way ANOVA, *p* = 0.015; Figure 4A).

### 3.4 Curcumin attenuated the inflammatory progression in the hippocampus of transgenic mice

**FIGURE 5:**
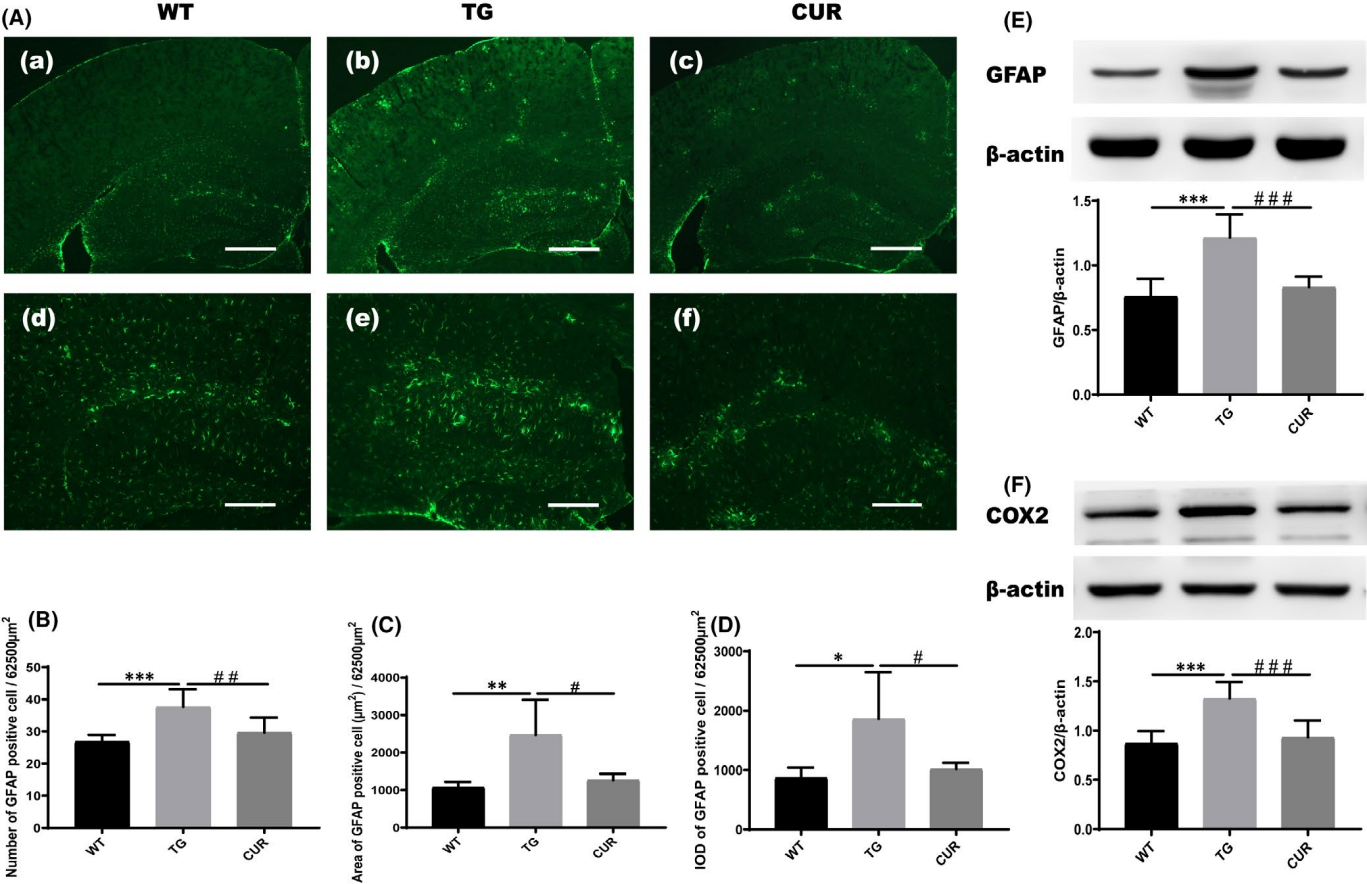
Effect of curcumin on inflammatory progression in the hippocampus of APPswe/PS1dE9 transgenic mice. (A) GFAP‐positive cell (green) detected by immunofluorescence. Scale bar represents 500 μm in a‐c and 200 μm in d‐f, respectively. (B) The GFAP‐positive cell counts of all mice. (C) The GFAP‐positive cell area of all mice. (D) The IOD of GFAP‐positive cell of all mice. (E) GFAP protein expression of all mice. (F) COX2 protein expression of all mice. WT, wild‐type mice; TG, control transgenic mice; CUR, curcumin‐treated transgenic mice. The data are presented as mean ± SD (*n* = 3), and rank‐sum test was performed for the area and IOD of GFAP‐positive cell and one‐way ANOVA for GFAP‐positive cell counts, protein expression, and COX2 protein expression. *p < 0.05 TG vs. WT; **p < 0.01 TG vs. WT; ***p < 0.001 TG vs. WT; ^#^ *p* < 0.05 CUR vs. TG; ^##^ *p* < 0.01 CUR vs. TG; ^###^ *p* < 0.001 CUR vs. TG

A low basal COX2 expression is present in neurons of the healthy brain; however, pro‐inflammatory conditions (such as astrocyte activation) strongly induce COX2 in the brain. To determine the anti‐inflammatory effects of curcumin, the expression of GFAP protein was visualized by immunofluorescence and Western blot analysis. Measurement of GFAP‐positive cell number and GFAP protein expression was used as the main index of astrocyte activation. Accordingly, curcumin treatment showed an anti‐inflammatory effect in immunofluorescence examination (Figure 5A). The results revealed that the TG mice tended to show more cell numbers (37.59 ± 5.57), larger area (2470.31 ± 938.41) and higher IOD (1862.82 ± 786.11) of GFAP‐positive cell compared with the WT group (cell numbers: 26.81 ± 2.18, one‐way ANOVA, *p* < 0.001, area: 1070.16 ± 149.48, rank‐sum test, *p* = 0.006, IOD: 870.12 ± 173.42, rank‐sum test, *p* = 0.015). Further, the mice in the CUR group displayed lower expression of GFAP compared with the TG mice, as indicated by fewer cell numbers (29.67 ± 4.71, one‐way ANOVA, *p* = 0.001), smaller area (1261.88 ± 173.54, rank‐sum test, *p* = 0.014) and lower IOD (1019.32 ± 102.14, rank‐sum test, *p* = 0.036) (Figure 5B‐D). Moreover, the protein expression of GFAP (1.21 ± 0.18) and COX2 (1.33 ± 0.17) was significantly increased in the TG mice compared with that in the WT group (GFAP: 0.87 ± 0.14, one‐way ANOVA, *p* < 0.001; COX2: 0.87 ± 0.12, one‐way ANOVA, *p* < 0.001), as detected by Western blot analysis. Curcumin treatment attenuated the protein expression of COX2 and GFAP, compared with that in TG mice (GFAP: 0.83 ± 0.08, one‐way ANOVA, *p* < 0.001; COX2: 0.93 ± 0.17, one‐way ANOVA, *p* < 0.001) (Figure 5E,F). We also evaluated the expression of CD68, an activated microglial marker. The evidence collectively demonstrated that microglial activity was attenuated in the curcumin‐treated group compared with transgenic mice (Figure S1A). These results indicate that curcumin attenuates some degree of neuroinflammation in the hippocampus of transgenic mice.

### 3.5 Curcumin decreased the expression of HMGB1, RAGE, TLR4 and NF‐κB p65 in the hippocampus of transgenic mice

**FIGURE 6:**
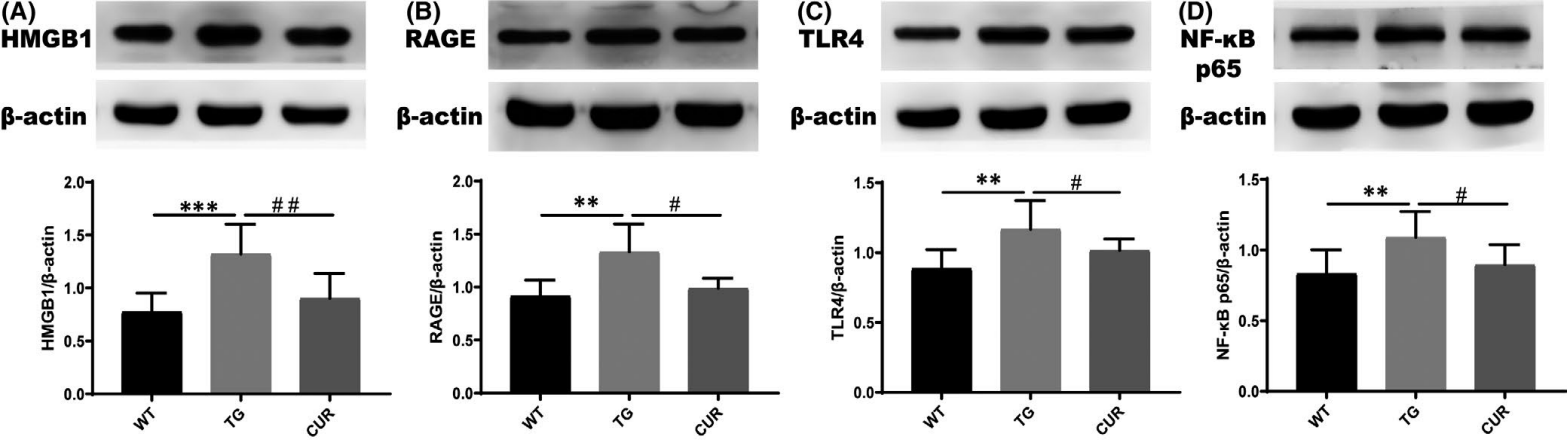
Effects of curcumin on the protein expression of inflammatory factor (A, B, C, D). WT, wild‐type mice; TG, transgenic control mice; CUR, curcumin‐treated transgenic mice. The data represent the mean ± SD (*n* = 4), and one‐way ANOVA was performed. **p < 0.01 TG vs. WT; ***p < 0.001 TG vs. WT; ^#^ *p* < 0.05 CUR vs. TG; ^##^ *p* < 0.01 CUR vs. TG

To investigate the signalling pathway of HMGB1/NF‐kB in the TG mice group, the expression of HMGB1, RAGE, TLR4 and NF‐κB p65 was examined by Western blot analysis (Figure 6). The protein levels of HMGB1, RAGE, TLR4 and NF‐κB p65 were significantly increased in the hippocampus of the TG group compared with WT (HMGB1: 1.32 ± 0.28 vs. 0.78 ± 0.18, *p* < 0.001; RAGE: 1.33 ± 0.27 vs. 0.92 ± 0.15, *p* = 0.004; TLR4: 1.17 ± 0.21 vs. 0.89 ± 0.14, *p* = 0.001; NF‐κB p65: 1.09 ± 0.18 vs. 0.83 ± 0.17, *p* = 0.003, one‐way ANOVA). Further, we explored the effect of curcumin on these proteins. Interestingly, oral administration of curcumin significantly attenuated HMGB1 (0.90 ± 0.23, one‐way ANOVA, *p* = 0.001) in the hippocampus of TG mice (Figure 6A). Similarly, curcumin also significantly decreased the receptors of HMGB1, including RAGE (0.99 ± 0.10, one‐way ANOVA, *p* = 0.013) (Figure 6B) and TLR4 (1.02 ± 0.08, one‐way ANOVA, *p* = 0.042) (Figure 6C). Moreover, curcumin significantly reversed the expression of NF‐κB p65 (0.90 ± 0.14, one‐way ANOVA, *p* = 0.02) in the hippocampus (Figure 6D). These figures indicate that curcumin decreased the expression of HMGB1, RAGE, TLR4 and NF‐κB p65 in the hippocampus of transgenic mice.

### 3.6 Curcumin treatment did not affect plaque load in the hippocampus of transgenic mice

**FIGURE 7:**
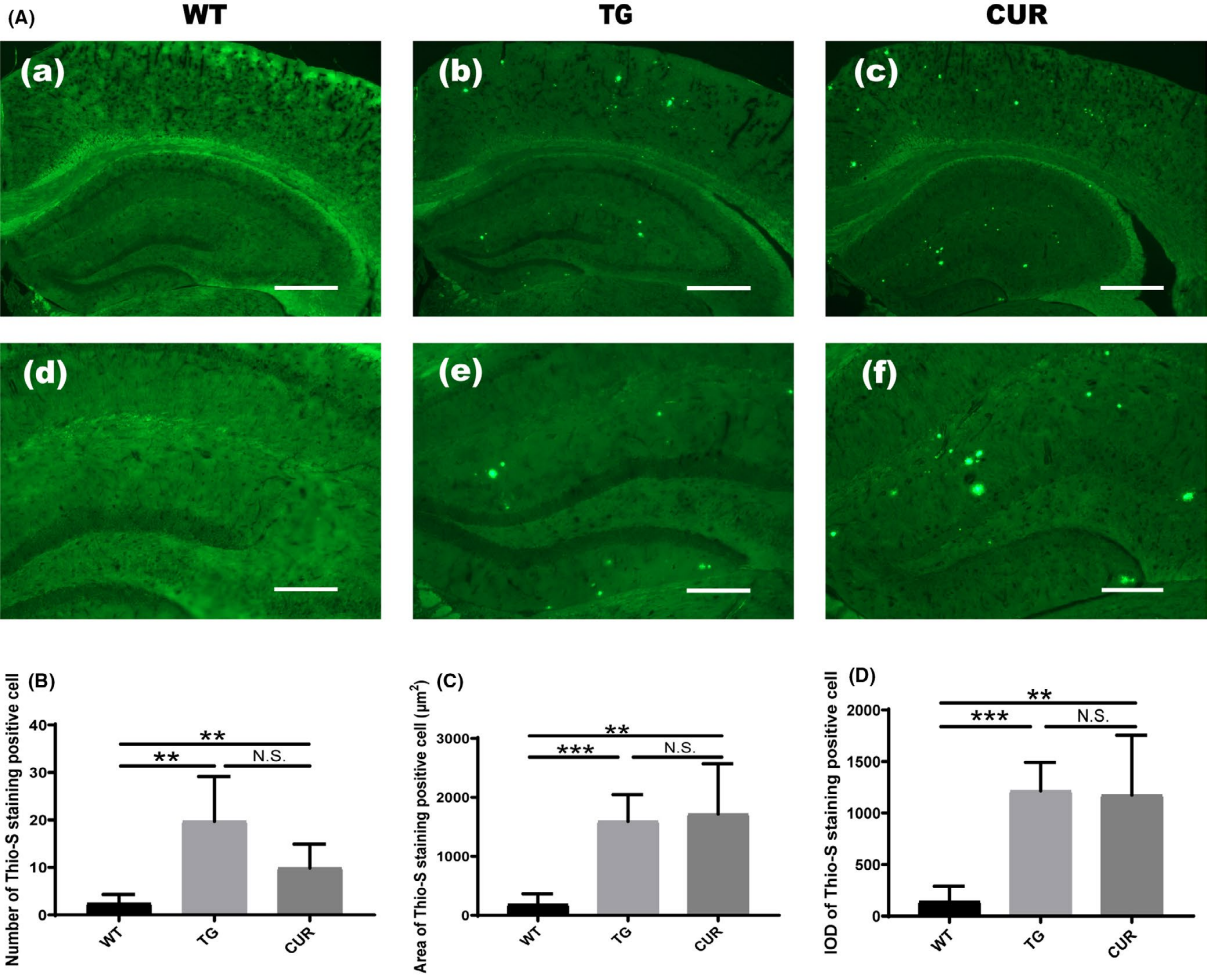
Effects of curcumin on plaque load in the hippocampus of APPswe/PS1dE9 transgenic mice. (A) Amyloid plaques (green) detected by thioflavin‐S staining of all mice. Scale bar represents 500μm in a‐c and 200μm in d‐f, respectively. (B) The thioflavin‐S staining positive cell counts of all mice. (C) The thioflavin‐S staining positive cell area of all mice. (D) The IOD of thioflavin‐S staining positive cell of all mice. WT, wild‐type mice; TG, control transgenic mice; CUR, curcumin‐treated transgenic mice. The data are expressed as mean ± SD (*n* = 3), and one‐way ANOVA was performed. **p < 0.01 TG vs. WT; ***p < 0.001 CUR vs. WT

In this study, thioflavin‐S staining was used to determine whether curcumin treatment affected hippocampal plaque load between experimental groups (Figure 7A). As shown in Figure 7B, the number of amyloid plaques in the hippocampus of the TG group (19.67 ± 9.47, one‐way ANOVA, *p* = 0.002) and CUR group (9.89 ± 5.01, one‐way ANOVA, *p* = 0.005) was higher than those in the WT group (2.44 ± 1.88). Similarly, the mean plaque area and IOD of thioflavin‐S staining amyloid plaques in the TG group (area: 1591.06 ± 457.35, *p* < 0.001; IOD: 1213.88 ± 276.47, *p* < 0.001) and CUR group (area: 1716.90 ± 853.44, one‐way ANOVA, *p* = 0.002; IOD: 1174.24 ± 580.80, one‐way ANOVA, *p* = 0.002) was also significantly higher than that in the WT group (area: 181.21 ± 183.21; IOD: 144.21 ± 147.43) (Figure 7C,D). Interestingly, there was no difference between the chronic curcumin treatment group and the TG group in terms of the number (one‐way ANOVA, *p* = 0.053), area (one‐way ANOVA, *p* = 0.974) and IOD (one‐way ANOVA, *p* = 0.997) of thioflavin‐S positive staining cells. The results showed that chronic curcumin treatment did not affect the plaque load in the hippocampus of transgenic mice.

## 4 DISCUSSION

The primary neuropathological feature of AD is the deposition of Aβ plaque, generated from the sequential cleavage of a transmembrane protein, the amyloid precursor protein (encoded by APP). Familial AD (fAD) results from gene mutations in the APP and presenilin (PSEN1 and PSEN2) genes.^1^ Our previous research focussed on microglia and neuronal cell interaction under Aβ_25‐35_ stimulation in vivo.^23^ This study further demonstrated the important role of Aβ plaques in the chronic neuroinflammatory features of AD mice. We found that several key proteins (including HMGB1, RAGE, TLR4, NF‐κB p65 and COX2) were altered during neuropathology. In the present study, thioflavin‐S staining revealed that amyloid plaques were increased significantly in APP/PS1 double transgenic mice, an ideal animal model to investigate the pathological process of AD. The results were consistent with previous studies that showed that nine‐month‐old transgenic mice exhibited memory deficits assessed by both MWM and Y‐maze tests, suggesting that there was a short‐term working memory decline in transgenic mice.^24^ Recent evidence indicates that AD is a neurodegenerative disorder in which chronic neuroinflammation contributes to disease escalation.^4^ Our results show that GFAP expression, often elevated in inflammatory conditions, was increased in the transgenic mice compared with that in the WT mice, as measured by immunofluorescent staining analysis.

Currently, no effective therapies are available to prevent or delay the AD pathogenesis progression.^2^ A recent study showed that aducanumab, a human monoclonal antibody that selectively targets aggregated Aβ, penetrates the brain‐blood barrier and decreases Aβ in patients with AD in a time‐ and dose‐dependent manner. This strategy is still being tested in phase 3 clinical trials and needs to be validated through larger studies.^25^ A polyphenol‐rich diet, which is more available and safer, can ameliorate cognitive disturbance.^26^ Curcumin is a natural polyphenolic product derived from the rhizome of *Curcuma longa* and has various beneficial properties, such as anti‐inflammatory, antioxidant and antitumor properties. Here, we found that curcumin treatment effectively improved the cognitive performance of transgenic mice in the MWM and Y‐maze, and our results were consistent with those of previous studies.^24^, ^27^ The cleavage of the APP pathway is divided into physiological and pathological pathways, with the latter resulting in Aβ production. Soluble Aβ_40_ and Aβ_42_ species are the predominant neurotoxic peptides in Aβ plaque.^28^ Mei et al. proved that total Aβ and Aβ_42_ peptide chains were reversed by curcumin treatment in the hippocampus through an ELISA.^29^ This evidence suggested that curcumin attenuated AD associated with cognitive decline, rather than soluble neurotoxic peptides in Aβ plaques. In this study, thioflavin‐S staining showed that curcumin treatment did not affect the plaque load in the hippocampus of transgenic mice. Nevertheless, the activation of astrocyte cells was suppressed by curcumin treatment in our study, which is in line with a previous report by Lim.^30^ A preliminary investigation of the possible mechanisms of curcumin action was performed. It is commonly considered that soluble Aβ oligomers, rather than monomers or plaques, may be the primary source of neurotoxicity.^31^, ^32^

The evidence suggests that HMGB1, which has a close relationship with the amyloid cascade, might be one of the most important signalling molecules in the pathogenesis of AD. HMGB1, a DNA‐binding nuclear protein, induces neurotic plaques independent of Aβ and inhibits the degradation of Aβ deposition. It is actively released following Aβ and cytokine stimulation, as well as passively during the death or autophagy process of neurons, microglia and astrocytes.^33^ The level of HMGB1 is significantly increased in the temporal cortices of the brain in AD patients^7^ and in those with other neurodegenerative conditions.^34^ HMGB1 and Aβ_42_ were found to co‐localize in senile plaques associated with microglia.^7^ Extracellular HMGB1 inhibits the degradation of Aβ_40_, delays the clearance of Aβ_42_, enhances the neurotoxicity of Aβ_42_ and accelerates neurodegeneration in an animal model of animals.^9^, ^35^

The HMGB1‐induced signalling pathway might be sustained as positive feedback or amplified at different levels. The interaction of HMGB1 with its cell surface receptors, such as RAGE and TLR4,^36^ mediates the phosphorylation of NF‐κB, which increases the production of cytokines and stimulates glia, ultimately leading to neurodegenerative disorders.^9^ Signalling through RAGE leads to activation of the NF‐κB pathway, as well as to signal transduction through extracellular regulated protein kinases (ERK) and p38 mitogen‐activated protein kinases (MAPK). In the HMGB1‐dependent activation of the TLR4‐signalling pathway, the myeloid differentiation primary response protein 88 (MyD88) is an important adapter protein of TLR4, subsequently regulated by the NF‐κB signalling.^37^ It has been demonstrated that the increase in pro‐inflammatory NF‐κB nuclear translocation in the region of the hippocampus, implicating synaptic pathology, exerts an effect on cognitive impairment.^22^ Mazarati^38^ showed that elevated levels of HMGB1 in the brain induced memory abnormalities, which may be mediated by both TLR4 and RAGE. Our previous results showed that HMGB1 gene silencing reduced RAGE and TLR4 expression, and decreased NF‐ĸB activity in vivo.^22^ In the present study, HMGB1, RAGE and TLR4 were upregulated in the hippocampus of nine‐month‐old APP/PS1 transgenic mice compared with WT mice. Additionally, NF‐κB p65, a subunit of the NF‐кB p50/p65 complex,^39^ was also upregulated in the hippocampus of transgenic mice.

The protective effect of curcumin may be mediated by the inhibition of NF‐κB activation^40^ and/or an antioxidant pathway.^27^ Oral administration of curcumin reduces inflammatory cytokines and oxidized proteins^30^, ^41^ and attenuates behavioural impairment in AD animal models.^42^ There is a low basal COX2 expression in neurons of the healthy brain; however, pathological conditions, such as astrocyte activation, strongly induce COX2 in the brain.^43^ The prevention of astrocyte activation and inhibition of HMGB1, RAGE and TLR4, as shown in our study, together with NF‐κB inhibition by *curcuma*, reported previously,^40^, ^44^ point to the fact that its anti‐inflammatory effects may play an important role in the development of AD. In clinical trials, curcumin appears to have no major side effects in patients with AD.^45^, ^46^

In conclusion, this study demonstrated that curcumin treatment significantly ameliorates cognitive impairment in aged APP/PS1 transgenic mice. The possible underlying mechanism might be associated with the accumulation of amyloid plaques, activation of the HMGB1‐RAGE/TLR4‐NF‐κB signalling pathway, and astrocytes activated during neuroinflammation in APP/PS1 transgenic mice. These results suggest that curcumin treatment, as a food additive for long‐term oral administration, is an effective therapeutic strategy for AD, although further studies are needed.

## CONFLICT OF INTEREST

The authors declare that they have no competing financial interests.

## AUTHOR CONTRIBUTIONS

**Yuan Han:** Conceptualization (equal); Investigation (lead); Writing‐original draft (lead). **Rui Chen:** Investigation (equal); Software (equal). **Qicheng Lin:** Investigation (equal); Visualization (equal). **Yu Liu:** Methodology (supporting); Software (equal). **Wenwei Ge:** Investigation (equal); Visualization (equal). **Hong Cao:** Conceptualization (lead); Project administration (equal); Writing‐review & editing (equal). **Jun Li:** Conceptualization (lead); Funding acquisition (lead); Project administration (equal); Writing‐review & editing (equal).

## Supporting information

Fig S1

## Data Availability

The authors confirm that the data supporting the findings of this study are available within the article.
