# Supplementary figures and images for: Curcumin improves memory deficits by inhibiting HMGB1‐RAGE/TLR4‐NF‐κB signalling pathway in APPswe/PS1dE9 transgenic mice hippocampus
Source: J Cell Mol Med. 2021 Aug 18;25(18):8947–56. doi: 10.1111/jcmm.16855 (PMC8435415; doi:10.1111/jcmm.16855)

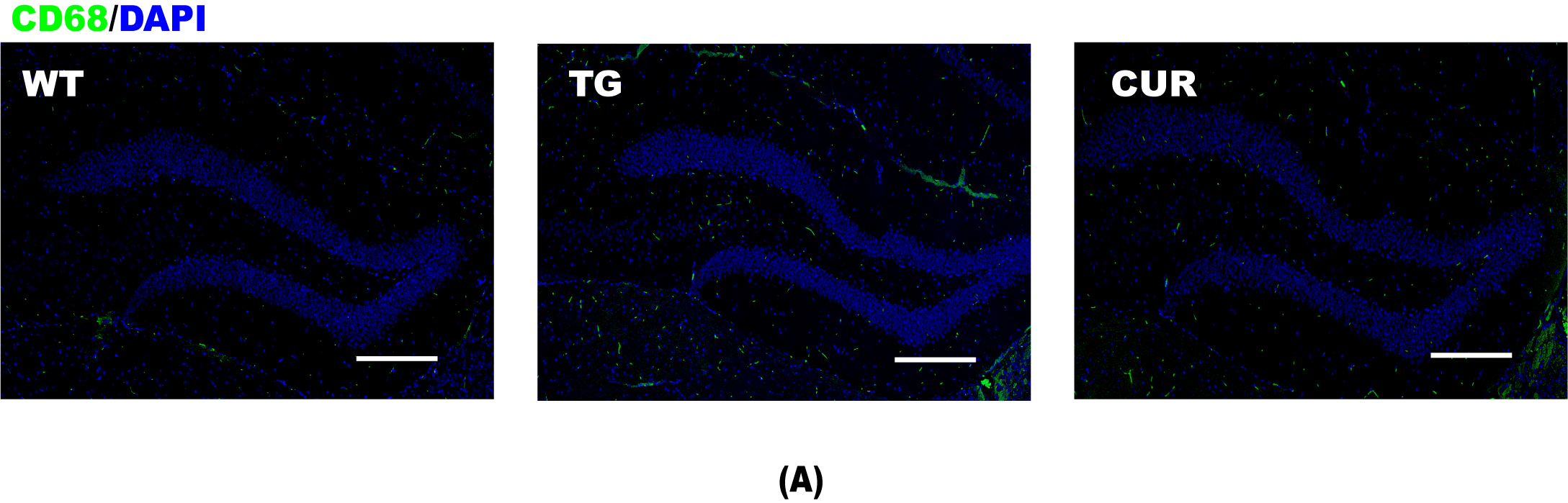

Supplement: Supplementary file 1 — Fig S1 [file JCMM-25-8947-s001.jpg]
